# Advances in the Methods for the Synthesis of Carbon Dots and Their Emerging Applications

**DOI:** 10.3390/polym13183190

**Published:** 2021-09-20

**Authors:** Areeba Khayal, Vinars Dawane, Mohammed A. Amin, Vineet Tirth, Virendra Kumar Yadav, Ali Algahtani, Samreen Heena Khan, Saiful Islam, Krishna Kumar Yadav, Byong-Hun Jeon

**Affiliations:** 1 Industrial Chemistry Section, Aligarh Muslim University, Aligarh 202002, India; areebakhayal12@gmail.com; 2 School of Environment and Sustainable Development, Central University of Gujarat, Gandhinagar 382030, India; vinars27dawane2009@gmail.com; 3 Department of Chemistry, College of Science, Taif University, Taif 21944, Saudi Arabia; mohamed@tu.edu.sa; 4 Mechanical Engineering Department, College of Engineering, King Khalid University, Abha 61411, Saudi Arabia or v.tirth@gmail.com (V.T.); alialgahtani@kku.edu.sa (A.A.); 5 Research Center for Advanced Materials Science (RCAMS), King Khalid University Guraiger, Abha 61413, Saudi Arabia; 6 School of Sciences, P. P. Savani University, Kosamba 394125, India; virendra.yadav@ppsu.ac.in; 7 Centre of Research and Development, YNC ENVIS PRIVATE LIMITED, New Delhi 110059, India; samreen.heena.khan@gmail.com; 8 Civil Engineering Department, College of Engineering, King Khalid University, Abha 61413, Saudi Arabia; sfakrul@kku.edu.sa; 9 Faculty of Science and Technology, Madhyanchal Professional University, Ratibad 462044, India; envirokrishna@gmail.com; 10 Department of Earth Resources and Environmental Engineering, Hanyang University, Seoul 04763, Korea

**Keywords:** carbon dots, electrochemical synthesis, energy storage, nanotechnology, graphene

## Abstract

Cutting-edge technologies are making inroads into new areas and this remarkable progress has been successfully influenced by the tiny level engineering of carbon dots technology, their synthesis advancement and impressive applications in the field of allied sciences. The advances of science and its conjugation with interdisciplinary fields emerged in carbon dots making, their controlled characterization and applications into faster, cheaper as well as more reliable products in various scientific domains. Thus, a new era in nanotechnology has developed into carbon dots technology. The understanding of the generation process, control on making processes and selected applications of carbon dots such as energy storage, environmental monitoring, catalysis, contaminates detections and complex environmental forensics, drug delivery, drug targeting and other biomedical applications, etc., are among the most promising applications of carbon dots and thus it is a prominent area of research today. In this regard, various types of carbon dot nanomaterials such as oxides, their composites and conjugations, etc., have been garnering significant attention due to their remarkable potential in this prominent area of energy, the environment and technology. Thus, the present paper highlights the role and importance of carbon dots, recent advancements in their synthesis methods, properties and emerging applications.

## 1. Introduction

Nanotechnology has played an immense role in each and every field of the sciences, whether in medicine [1,2], research or environmental cleanup. No doubt, nanotechnology and NPs have played none other than a second role in the 21st century. All these areas of science have widely exploited the metal and metal oxide nanoparticles, but such NPs have numerous drawbacks such as their non-biodegradable nature, toxicity, etc. When such NPs are surface-functionalized, then their acceptance increases and manifests in various fields. In medicine, NPs, with altered biophysical attributes, are being used to make incredibly spreadable, biocompatible nano pharmaceuticals with lower immunity and greater protection, including enhanced solubility [3,4]. 

Since ancient times, several metallic and nonmetallic particles have been used to control the growth of microorganisms, especially in agricultural fields to protect the crop from various bacterial and fungal diseases. Meanwhile, pesticides and insecticides were also used very frequently, which led to toxicity for human beings. Insecticides are often used to combat plant diseases, while constant use always creates serious environmental problems. So far, much effort has gone into the development of antibiotics, drugs and antibacterial ingredients. A large number of nanoparticles (for example, cationic conjugated polymers, dehydrogel peptides, semiconductors, noble metal particles, graphene layers with carbon tubes, graphene oxide and element-doped carbon nanoparticles) have been used, increasing the permeability of bacterial membranes; they are conventional in comparison with antibiotics [5]. Such antibacterial nanomaterials are still rich in microbial resistance and particle deterioration [6], resulting in a slew of alternative pathogens or even risks. 

Constantly degrading nanomaterials could impact the taxonomic category affiliated with the analysis in a defined N_2_ ecosystem, methane oxidation and complex decomposition by modifying the proportion of microbial groups in a therapeutic dose [7]. It would be desirable to develop biologically safe and biodegradable nanomaterials with various antibacterial properties. So, there was a need for biocompatible and less toxic materials for this application, and carbon dots were found to be most suitable. 

So, carbon particles, especially carbon nanomaterials [8], have gained great attention, not only due to their biodegradable and less toxic nature, but also due to their easy surface functionalization. Recently, carbon dots (CD) have been useful, plentiful and low cost [9], and have steadily become the performer throughout the nanocarbon dot community, prompting comprehensive research owing to high opportunity for a variety of technological implementations [10]. Carbon quantum dots (CQDs) are a novel fluorescent nanostructure comprising a carbon network formed via an indistinct carbon framework. Such particles have low toxicity, strong fluorescence, tunable color emission, easy surface functionalization, low cost, high aqueous stability and photostability [11]. Various approaches have been used to synthesize CQDs such as (i) top-down pathways, such as the laser ablation technique [12], electrochemical exfoliation (ECE) [13] and the chemical oxidation method (destruction of large carbon materials) [14], or (ii) bottom-up pathways (small precursor composition): microwave-associated synthesis [15], plasma [16] and hydrothermal and solvothermal treatments [17]. CQD has been recorded to reduce antimicrobial properties and potency based on the scale including charge density, which could be conveniently regulated by surface modification (for example -COOH, -OH, amino, epoxy, NH_2_, etc.) [17]. CQD causes disintegration of the membrane as well as the generation of reactive oxygen species (ROS); both are suitable research areas for antimicrobial action [18]. Enhanced ROS levels and oxidative exposure alter genetic material as well as the protein shape, and CQD is compatible with graphene oxide, single-wall carbon nanotubes and fullerenes, although it is seldom reported with other carbon nanostructures [5]. Carbon nanomaterials’ antimicrobial effects [19] have been thoroughly researched, and further comprehensive research is required to clarify the interaction between surface chemistry, CQD and bactericidal effects [20]. Meanwhile, fluorescent carbon (CD) dots with size parameters, water solubility, lightfastness, especially high levels of biocompatibility and low toxicity have generated worldwide interest in disciplines such as bioimaging, analysis and gene therapy [20]. According to such properties, this new kind of nanoparticle (NP) outperforms quantum dots (high toxicity problems) [21] and conventional dyes (photobleaching) [22]. The most recent application of CDs is in the field of chemical warfare agents, especially for the detection of hazardous chemicals. These CDs were used as either functionalized or non-surface modified forms, which was reported by Kumar et al., Paul et al., Tuccitto et al., Tuccitto et al., Tuccitto et al. and Butero et al. [23,24,25,26,27,28].

The current review highlights the various synthesis approaches and their applications in fields such as chemical warfare agents, medicine, environmental cleanup, etc. The objective of this study is to provide state-of-the-art research into the synthesis and applications of carbon dots in recent years.

## 2. Synthesis of Carbon Dots

Carbon dots (CDs) are similar to quantum dots, where the source of precursor material is mainly carbon in the case of carbon dots, whereas the sources of quantum dots are metal oxides, metal sulfide, etc. QDs are sometimes known as “artificial atoms”, which are actually nanocrystals with very small sizes. They have acquired the name “artificial atoms” due to the possession of quantum effects similar to conventional atoms, which is linked with the fact that they are “tailor-made materials” [29]. CDs can be classified into three basic types: graphene quantum dots, carbon quantum dots and carbonized polymer dots.

**Figure 1:**
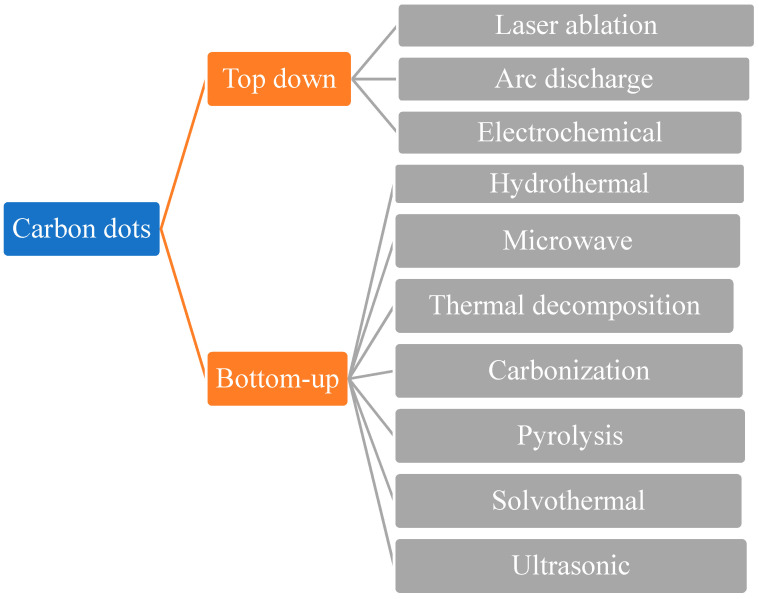
Different methods for the synthesis of carbon dots.

**Figure 2:**
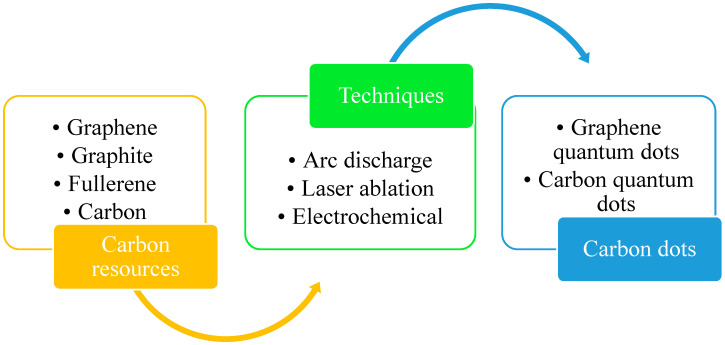
Top-down approach for the synthesis of carbon dots.

Top-down and bottom-up synthetic approaches to C-dots seem to be the most prominent. Laser ablation (LA), arc discharge (AcD), electrochemical techniques and plasma treatment are all forms of top-down methods for cutting carbon materials using carbon nanomaterials [30]. Pyrolytic processes, template methods, supported synthetic strategies, microwave-based methods, chemical oxidation, reverse micelle processes and others are instances of bottom-up methodologies [31]. All these top-down and bottom-up approaches for the synthesis of C-dots are shown below in Figure 1. Top-down approaches for manufacturing C-dots are shown in Figure 2.

### 2.1. Laser Ablation (LA)

LA is a synthetic method that involves the use of a laser and a carbon source for the synthesis of carbon dots. Sun et al. used a method to generate fluorescent C-dot, where they first mixed graphite powder and cement and then heat-treated it to obtain a carbon source. Furthermore, by using a laser source, carbon was removed from the surface by using an argon gas vapor stream at 900 °C and 75 kPa to obtain carbon nanoparticles (CNPs) [32]. Such synthesized CNP sizes were variable, which exhibited no photoluminescence (PL). The sample was then treated with polyethylene glycol (PEG1500N) or poly (propionyl ethylene-imine-ethyleneimine) after being refluxed in aqueous nitric acid over 12 h (PPEI-EI). The PL of passivated C-dots with a diameter at about 5 nm was very powerful, whose fluorescence quantum yields ranged from ~4% to 10% at 400 nm excitation [33,34,35].

Likewise, Gonsalves et al. produced carbon nanoparticles by embolization immediately from a carbon target dissolved in non-ionized liquid; the carbon particles that resulted would not fluoresce [36]. The carbon nanomaterials were deposited in aqueous nitric acid and then allowed to stand for 12 h to enable the carbon nanoparticles’ surface, after which PEG200 was applied to the mixture and boiled under reflux for 28 h, followed by mercapto-succinic acid and warmed for 31 h. Then, the colorless mixture turned to light brown, fluorescent C-dots via a size distribution of 267 nm. The scale of the poly ethyl glycol (PEG) chain as well as the existence of other parameters had no impact on the long-term destruction of the activated C-dot. Iodide reduces the fluorescence strength of the C-dot; however, other metals including Hg^2+^, Ca^2+^, Ni^2+^, Zn^2+^, Cd^2+^, Zn^2+^ and Cu^2+^ ions disperse. Therefore, it has no impact on the fluorescence of C-dots, so it could be employed to monitor iodine [36].

By dispersing ultrasonic laser radiation throughout a PEG1500N mixture over 4 h, a uniform black suspension was produced. C-dots were achieved from a color precipitate following centrifugation (5000 rpm). The average thickness of the C-dot microstructure could affect quantum fluorescence performance, which could be regulated by changing the pulse width of a laser pulse [37]. Laser ablation has a number of advantages, including ease of use and the ability to produce a variety of nanostructures. However, in order to meet the carbon mark, this approach necessitates a large amount of carbon materials [38]. Carbon nanostructures produced by laser radiation have a wide range of sizes, and large particles could be conveniently separated by centrifugation, which leads to effective utilization of carbon nanoparticles and carbon materials [39]. Calabro et al. synthesized carbon quantum dots and conducted a comparative analysis. They used a one-step pulse ablation process and chemical oxidation of carbon nano-onions to make graphene quantum dots. The authors compared the photoluminescence spectra of both quantum dots and concluded that the laser ablation model seemed to have a blue shifted emission compared to the chemical oxidation one that they referred to in the particles scale and surface functional group impact. The authors also emphasized that the laser ablation process product had a stronger decreased thickness and an estimated diameter of single coat about 1.8(6) nm, whereas the chemical oxidation product had an approximate dimension of 4.1(8) nm and a thickness compared to 2–3 graphene layers. The authors also elaborated functional group preferences as well as the carbon friction phenomenon which was different for both cases, such as the fact that the oxidation of chemical quantum dots favored carboxylic categories or a larger sp^2^ carbon content although laser ablation quantum dots supported hydroxyl categories as well as a larger sp^3^ carbon content. The authors used the PL longevity observations to demonstrate important insights into the radiative as well as non-radiative decay pathways of the functional groups (FG) at the surface and sp^2^ carbon domains, which were reported to be 7.9(6) ns for emission by –COOH categories and 3.18(10) ns for emission by hydroxyl categories, accordingly. In comparison to chemical oxidation, the authors concluded that liquid form laser ablation produces quantum dots in a much faster and safer one-step procedure with fewer initial chemicals and residues [40,41]. The various studies carried out for the synthesis of C-dots by laser ablation technique are given below in Table 1.

### 2.2. Arc Discharge (AcD)

Xu and colleagues oxidized AD soot using 3.3 M nitric acid to introduce the –COOH group; after that, they extracted the chamber with the NaOH method of producing a strong black slurry [47]. The fast-moving band is distinguished from C-dots, which seem to be extremely fluorescent and have an approximate dimension of around 18 nm during gel electrophoresis [49]. Bottini et al. used the electronic flash method to isolate PL nanomaterials from clean and nitric-acid-oxidized carbon nanostructures [50,51]. The hydrophobic, narrow-spreading carbon nanotube luminescent nanomaterials appeared impeccable. When oxidized carbon tubes were distributed in water, fluorescent nanomaterials could accumulate. Arc discharge created a very small amount of carbon nanomaterials. AcD dust often includes a number of complicated materials that are hard to extract. The various studies carried out for the synthesis of C-dots by the arc discharge technique are given below in Table 2.

### 2.3. Electrochemical Approach

Lou et al. used a 2 cm distance between a high-purity graphite rod including strongly focused pyrolytic graphite as well as a platinum (Pt) wire as an anode and a counter electrode [55]. After that, they immersed it in a liquid/water ionic mixture, and then assigned persistent ability to encourage the carbon content to peel away. The peeling procedure comprised a diverse relationship between anionic fluid inserted and anodized water isolation, filter isolation and ultracentrifugation, which were being used to acquire 6 nm scale C-dots at 15,000 rpm at 20 °C from water poured over the exfoliation compounds including ethanol prior to pH neutralization; fluorescent quantity yield was about 2.8–5.2 percent. In the study by Yao et al., in the center, a pure graphite loop electrode as well as a titanium tube cathode was assembled [56].

An insulative O-ring had separated the anode and the cathode and electrolyte were made of pure water. Electronic voltage and ultrasonic control were used; therefore, bright blue fluorescent C-dots were widely utilized in distilled water with no need for complicated cleansing. The scale of the synthesized C-dots was around 2–3 nm and the quantity yield was 8.9%. In the aqueous phase, the C-dots provided excellent fluorescent characteristics as well as thermodynamic consistency [56]. Naik et al. synthesized nitrogen-doped carbon dots by using the hydrothermal process and made a decent naked eye fluorescence probe for dopamine sensing [57]. They obtained a linear range of 2–20 μg/mL along with a detection limit as low as 1.97 μg/mL. Qin et al. used a one-step hydrothermal synthesis method for the preparation of novel carbon dots from *Escherichia coli* BW25113 (WT), which were found to be more useful in microbial imaging [58]. The synthesized carbon dots were found to be applicable in live as well as dead microbial imaging along with significant attributes such as biocompatibility, excellent penetrability and nontoxic characteristics such as fluorescent probes in both in vitro and in vivo microbial imaging applications. Edison et al. used a hydrothermal synthesis process to create a nitrogen-rich blue fluorescent carbon dot (NR-CDs). They used citric acid monohydrate (CA) and 2-aminopyridine (2-AP) in this process of hydrothermal treatment [59].

Zhang et al. used one-pot hydrothermal synthesis for dual emission carbon dot preparation. During this hydrothermal treatment they used sodium citrate (SC), triethylenetetramine (TETA) and Rose Bengal (RB) as raw materials. In the typical synthesis, the hydrothermal environment not only helped sodium citrate and TETA to form an amide bond polymer similar to the intermediate but also initiated the halogen atom removal of the RB molecule that finally built a skeleton structure of the network intermediate. Further dehydration and carbonization resulted in the dual-emission characteristics [60,61]. Several studies carried out for the synthesis of C-dots by the electrochemical technique are given below in Table 3.

### 2.4. Microwave-Assisted Synthesis

Microwave-assisted synthesis is a simple and cost-effective process for synthesizing CDs by irradiating electromagnetic radiation with wavelengths ranging from 1 mm to 1 m by the reaction mixture of the precursor molecules [68]. Zhu et al. used first-time microwave irradiation to make fluorescent CDs with a size of ~3.7 nm. They heated saccharides and polyethylene glycol aqueous solution in a domestic microwave oven (500 W) for almost 3 min [69,70]. Liu et al. used glycerol as the synthesized multicolor photoluminescence CDs with an average size of ~5 nm carbon source and, as passivating agent, 4, 7, 10-trioxa-1,13-tridecanediamine (TTDDA) [71,72]. Wang et al. made water-soluble CDs with pyrolysis of citric acid assisted by a single-step microwave. They both used tryptophan (Tarp) as a passivating agent and as a source of nitrogen. They heated the aqueous solution of citrate and L-Tarp for 3 min in a microwave oven (700 W) and removed the large particles at 10,000 rpm by centrifugation to obtain CDs of ~2.6 nm [73]. Kiran et al. utilized citric acid as a carbon source and boronic acid with 3-aminophenyl as a passivation agent for CD manufacture [72,74]. In a microwave oven (1200 W), they heated the aqueous solution of citric acid and 3-aminophenyl boronic acid for 4 min and the average diameter of the CDs achieved ranged from 2 to 5 nm. Cao et al. recently reported CDs from aqueous glucose and arginine solution using microwave-assisted pyrolysis in a microwave oven (700 W) for approximately 10 min. The mean, as obtained, and the diameter of the CD were between 1 and 7 nm. Furthermore, several other researchers published on the microwave-assisted CD synthesis [75]. Several studies carried out for the synthesis of C-dots by the microwave-assisted technique are given below in Table 4.

### 2.5. Thermal Decomposition

The thermal decomposition technique has also been used by researchers as another traditional bottom-up approach to synthesize CDs. A material or compound is chemically decomposed in ordinary thermal decomposition by heat action. The thermal reactions to decomposition are typically endothermic [17]. Either this type of decomposition reaction is irreversible (decomposition of starch, protein) or reversible (ammonium chloride decomposition, calcium carbonate). The advantages of this method include ease of use, less time taking, cheap price and large-scale manufacturing [84].

Wang et al. reported highly luminescent CDs as the passivation agent by the thermal decomposition of citric acid as the source of carbon and organ silane, N-(*β*-aminoethyl)-γ-aminopropyl methyl dimethoxysilane (AEAPMS) [85]. They only heated the reaction mixture for 1 min at 240 °C and the observed CD diameter was ~0.9 nm. Wang et al., after that, produced the CDs from citric acid by using this method. They heated citric acid for 30 min on a hot plate at 200 °C, neutralized it with a solution of sodium hydroxide and finally solubilized it for purification. The size of CDs within the 0.7 to 1 nm range was observed. These CDs demonstrate independent photoluminescent (PL) and excitation-dependent properties, as well as differing QY depending on different conditions for synthesis. Wan et al. used the decomposition thermal method of 1-butyl 3-methyl bromide imidazolium and l-cysteine for CD fabrication at 240 °C. The AFM study showed that the height of the CDs ranged between 1.0 and 3.5 nm. Several other studies also found this method for determination of CDs from small organic molecules [85]. Several studies carried out for the synthesis of C-dots by thermal decomposition technique are given below in Table 5.

### 2.6. Carbonization Synthesis

Various precursor molecules can be carbonized easily; thus, this is one of the cheapest, recognized, convenient and superfast single-step techniques for CD manufacturing. Carbonization is a chemical process wherein, through continuous pyrolysis in an inert environment, solid materials with higher carbon content are formed from organic materials. Wei et al. used this superfast carbonization technique to produce N-doped CDs. They used a method that is much faster (2 min only) than glucose and ethylene-dia-amine as a source for carbon and nitrogen, respectively [94]. The scale of the prepared carbon dot was observed to be between 1 and 7 nm, along with 48% of QY. In a very remarkable work, Wang et al. produced blue-luminescence-producing carbon dots that were thermally reduced along with a 4.8 to 9 nm range in the size by using carbonization in the presence of citric acid in the medium. The thermal decrease phenomenon of carbon dots was evaluated by a thermogravimetric analyzer, leading to a five-fold increase in QY compared to non-reduced CDs. In another immense effort, Dolai et al. prepared nanoparticles in the ~2.4 ± 0.5 nm range by using 6-O-(O-dilauroyl-tartaryl)-D-glucose as the source of carbon in the synthesis medium [95]. Several studies carried out for the synthesis of C-dots by carbonization technique are given below in Table 6.

### 2.7. Pyrolysis Synthesis Method

Some researchers still favor the pyrolysis approach for synthesizing CDs from precursor molecules. Pyrolysis is also a type of thermal deposition method, but it is almost irreversible in nature, in which various samples or organic materials have been subjected to decomposition under inert conditions. Under this inert atmosphere, the physical and chemical changes occur in organic sample materials which lead to carbon-containing solid residues. Thus, pyrolysis usually uses controlled pressure and very high temperatures during the process [98].

In a faithful attempt under the 250 °C pyrolysis process, Bourlinos et al. fabricated a Gd (III)-doped carbon dot with the size range of ~3.2 nm with the remarkable properties of dual fluorescence [99]. They used a combination of a Tris base, gadopentetic acid and betaine hydrochloride to harvest Gd (III) CDs. Zheng et al. used a stepped forward pyrolysis method to synthesize a special category of carbon dots. L-aspartic acid and D-glucose were used as the reference molecules in this study. They reported 2.28 ± 0.42 nm size of prepared material by using an aqueous NaOH medium containing glucose and aspartic acid under the 200 °C temperature for 20 min. In another work, Feng et al. used a thermal pyrolysis method to prepare carbon dots of citric acid. They used diethyl-ene-tri-amine during this process and prepared 5–8 nm sized carbon dots by transmission electron microscope (TEM) evaluation [100,101]. Several studies carried out for the synthesis of C-dots by pyrolysis technique are given below in Table 7.

### 2.8. Solvothermal Method

The literature is replete with several studies related to the use of small organic molecules as the source of carbon to synthesize the carbon dots. Zhang et al., for instance, used carbon tetra chloride as a carbon source to make N-doped carbon dots with NaNH_2_ as a source of nitrogen via the solvothermal method [103]. As a result, a remarkable carbon dot was prepared with a size of 3.3 nm along with a 0.5–5 nm height. The authors prepared a very crystalline product by using the solvothermal method that showed a graphite-like structure. In another work in solvothermal synthesis, Qian et al. used SiCl_4_ and hydroquinone to produce Si-doped CDs. In a typical stainless-steel autoclave, the authors used acetone as solvent and made a mixture of SiCl_4_ and hydroquinone mixture within it and provided the heat at 200 °C for 120 min. As a result, they prepared a Si-doped carbon dots product with a size of 7 ± 2 nm [104]. Shan et al. used a solvothermal single-pot preparation process to develop boron-doped carbon dots. They included carbon precursor hydroquinone and boron source BBr_3_. As a result, they produced a B-doped carbon dots product with a size around ~16 nm. Several studies carried out for the synthesis of C-dots by hydrothermal and solvothermal techniques are given below in Table 8.

### 2.9. Ultrasonic Treatment

Ultrasonic has also been identified as an efficient technique to develop various carbon dots; thus, much literature has been associated with the synthesis of carbon dots by this method. In this technique, carbon precursors along with acid, alkali and other oxidants are kept under high ultrasound waves, due to which there is a breakage of carbon particles into very small nanoparticles. There is continuous cavitation of the molecules. The use of high energy of ultrasonic waves avoids the complex post-treatment process, thereby realizing the facile synthesis of CQDs with a small size [115,116,117]. In a very comprehensive work, Li et al. prepared a fluorescent carbon dot along with the ability of water solubility. They used activated carbon, employing the ultrasonic treatment approach, assisted by one-step H_2_O_2_ in the same year. The TEM findings showed that the surface of prepared carbon dots was rich in hydroxyl groups along with a detected size range of 5–10 nm [117]. Kumar et al. reported the synthesis of carbon quantum dots (CQDs) by using the sonochemical method and also described the detailed applications of CQDs in electronics. Pan et al. reported the synthesis of fluorescent CQDs by using the sonochemical method and applied them for sensing of food analysis [115,118]. Ultrasonication-based CQD synthesis was first reported in 2011 by Li and his fellow members, using glucose in an acidic and basic environment. Li and his colleagues synthesized PL CQDs, of sizes below 5 nm [117]. Huang and Lu et al. reported the synthesis of fluorescent CQDs by the ultrasonication method functionalized with thiol-terminated polyethylene glycol. Here, due to the addition of a hydrophobic PEG group, the dispersibility of the CQDs increased in the aqueous phase. Moreover, it also increased the biocompatible nature of the synthesized CQDS [119]. Lu et al. synthesized a blue, fluorescent N-CQD (size: 2.5–5.5 nm, QY: 3.6%, average life: 3.02 ns) by ultrasonic treatment of dopamine in dimethylformamide (DMF), which exhibited good stability of colloid and light in aqueous solution [119,120]. Several studies carried out for the synthesis of C-dots by the sonochemical technique are given below in Table 9.

## 3. Properties of CQDs

The emerging new class of carbon nanomaterials has unique and remarkable properties, due to which it has drawn the attention of the whole scientific community. The CODs have unique physical, chemical and optical properties. The chemical property of CQD is highly important among all the properties due to its photoluminescence phenomenon, chirality and UV absorption [122]. Typically, CDs can act as both electron acceptors and donors [123]. All these properties are discussed below in detail by citing relevant literature.

### 3.1. Structural Properties of C-Dots

Carbon dots (C-dots) are a novel group of nanomaterials that contain carbon and whose size is generally below 10 nm in size. Sun et al. claimed that quasi-spherical nanoparticles are carbon dots [124]. They have a diameter of less than 10 nm and some carbon dots are also hollow structured. Many researchers have already shown amorphous C-dots with geometry such as sp^2^ and some have been identified as sp^3^, in a diamond-like structure. The C-dots are circular or elliptical in shape, and others also have a quadrate, triangular and hexagonal structure, verified by high-resolution transmission electron microscope (TEM), tools for diffraction by scanning electron microscope (SEM) and X-ray diffraction (XRD) [124].

### 3.2. Absorbance of C-Dots

Due to the transition pi-pi* (π–π*) of double bonds (C=C), C-dots absorb in the short-wavelength region. Within the UV region (260–320 nm), they usually display heavy optical absorption, with a tail reaching up to the visible range [125]. C-dots are usually more effective at processing long wavelengths. Based on the surface passivation and the functional groups attached to its surface, their absorption properties vary from one C-dot to another [126].

## 4. Mechanisms Involved in the Photoluminescence Phenomenon

The mechanism of the photoluminescence of carbon dots is still widely debated, with the work focusing on finding the origin of photoluminescence-dependency on excitation [127]. The search is exceptionally challenging due to the large diversity of synthesis methods and precursors which influence both the assembly and configuration of the CDs. The description suggested can be summed up in three key mechanisms:

The effect of the quantum captivity or the phenomenon associated with core emission caused by the conjugated carbon core π-domains; surface states due to the presence of carbon-backbone-linked functional assemblies and molecular state in which the emission derives through free fluorescent or bound molecules.

### 4.1. The Core Emission

There is a direct link between the emission phenomenon that is generated because of excitation and the nanometer-level dimension of the particles in the exploration of carbon dots, seeking a special effect that is called the quantum confinement effect (QCE) on the optical functionalities as for the various quantum dots [128]. Several publications have investigated evident indications for QCE, due primarily to crystalline carbon core (CQD) observations.

In top-down synthesized CDs, Kang et al. recorded the significant red shift of emission from the ultraviolet zone to near infrared by increasing the dimension of carbon dots from 1.2 to 3.8 nm and associating the effect with the nanoparticles’ graphite-like structures at a quantum size [129]. Lately, the solvothermal framework enables the growth of crystalline carbon dots with related characteristics, requiring QCE, miraculously associated with surface oxidation [129].

The states among the carbon cores engage in the PL of carbon dots by recombining radiative excitons in the center arising from the pi-pi* (π–π*) transition in the sp^2^ constellations supported by the phenomenon related to quantum confinement [128]. The core emission is mainly comparative at tinier wavelengths and has a little PL QY; however, the existence of graphic nitrogen enables changes in the absorption, such as red shifting of absorption as well as variations in the emission-related properties, even after once again referring to the core construction role, and besides the oxidation phase, it has been expected to enhance the sp^3^ shell region and decrease the sp^2^ core structures that are further perturbed by N-doping, inducing emissive core trap states to form [47].

### 4.2. The Surface States

The surface states phenomena are the relatively most standard explanation for the peculiar carbon dots emission, associated with pH-related variations, solvatochromic consequences, effects related to oxidation and topological functionalization [130]. The intrinsic and extrinsic surface centers can be differentiated by altering the narrative of semiconductor fields. Intrinsic surface conditions are because of the dissolution of the underlying lattice of the particle, while extrinsic centers are surface lattice deficiencies as a result of adsorbed or bonded compounds. Most of these are usually accountable for the outcomes related to the emission surface states, at which the carbon backbone hybridization and the connected chemical groups greatly affect the various levels of electronic dynamism. The excitation dependence of the emission has been associated with the oxidation processes involved on the surface by correlating the red-shift emission with intensification in the oxidation of the surface [131]. Through means of quantum chemical measurements, the alteration of the electronic atmosphere through oxidation on sp^2^ carbons was demonstrated as the cause of the electronic emission rates in designed graphene oxide [132].

Sun et al. observed that when using organic molecules, surface passivation creates constant superficial energy set-ups that contribute to the PL emission [132]. The effect of multiple atoms on the surface, particularly different nitrogen molecules, adjusted the release features of carbon dots, attributing the blue color discharge to pyrrolic N_2_, the green color to pyridinic N_2_ and the red PL to p-phenylenediamine alteration. Thus, the various colored discharges such as blue, green and yellow were associated with surface conditions due to pH variations and solvent properties in hydroquinone- and EDA-prepared CDs. An XPS study found emissions were linked to surface-based imine groups and produced energy levels associated with defects [128].

### 4.3. The Molecular State

The basis of the model associated with the molecular state is the existence of the molecular materials that are fluorescent in nature, free or associated with the carbon dots’ structure. Various organic molecules have been categorized; (imidazo[1,2-α]pyridine-7-carboxylic acid (IPCA), 1,2,3,5-tetrahydro-5-oxo-7-indolizinecarbaldehyde and 4-hydroxy-1H-pyrrolo[3,4-c]pyridine-1,3,6(2H,5H)-trione (HPPT) have been accepted as accountable for blue (IPCA) and green (HPPT) emissions in bottom-up synthesis related with citric acid synthesis [74].

Excitation (the Kasha–Vavilov rule) has certain requirements. Therefore, supplementary appliances and/or the existence of a collection of discharging molecules are needed. The full spectral attributes of CDs can be effectively clarified by polycyclic aromatic hydrocarbons (PAHs). Thus, carbon dots can be called nanocrystals at molecular levels, where these polyaromatic hydrocarbons have been trapped in a sp^3^-hybridized carbon background. Because of their numerous band gaps, a collection of stacked PAHs, including perylene, anthracene and pyrene, may provide for the reliance on release excitation phenomenon [133].

Since then, Righetto et al. have reported permitted molecules using a coupling fluorescence correlation spectroscopy technique and paramagnetic resonance of time-resolved electrons [134]. However, on the other hand, the molecules that are bound to or incorporated within the layers, and the recorded oscillations and turnings of carbon layers, can provide for the expected hydrodynamic radius at molecular levels. Moreover, fluorophore materials have also been suggested to perform a part in the absorption and emission characteristics. CDs can be interpreted as synthetic objects, made from fluorophores, ultimately aggregated, within or connected to a carbonized heart, including these results [128]. That will further confirm the image, and it has been shown whether temperature and synthesis time can alter the molecular state/core balance. Several alternative approaches have been suggested to elucidate the development of carbon dots. There is the process involved with the hydrothermal phenomenon of CA and EDA, involving specific carbonization and controlled carbonization of involved molecules, thereby promoting the creation of a mixture of diverse emission sites in similar carbon dots or the creation of a dissimilar variety of diverse carbon dots characterized by their separate emissions [135]. The crosslink-enhanced PL system for polymer dots (PDs) was designed by dehydration, condensation, carbonization or assembly regions by non-conjugated polymers [136]. As such, PDs display an excitation-dependent emission characteristic attributed to the contribution of the centers that are fluorescent in nature, as well as chemically amine-based rotating and vibrating gestures, which are inhibited by the polymeric cross-linked frames. From the perspective of solid-state applications, surface states can also be implicated in engineering the secretion from carbon dots while assisting in hosting environments, such as silica media or zinc oxides.

### 4.4. Factors Affecting the Properties of C-Dots

There are several factors which govern the properties of carbon dots, for instance reaction temperature and time, pH of the synthesis conditions, type of precursor material used and amount of carbon present in them. The properties of C-dots also depend on the heteroatom codoping of the C-dots and surface passivation. The source of carbon dots generally affects the fluorescence property of the C-dots, as in one study, carbon dots prepared from pineapple peels were completely degraded within a few days due to fungal activity, while C-dots prepared from cucumber and peels showed better stability and no fungal degradation was observed. Synthesis of CDs involves carbonization, which is an endothermic process, so temperature plays a key role in the synthesis of carbon dots. An experiment conducted on various types of plant leaves used pyrolysis methods to obtain carbon dots. It was found that, though similar pyrolysis conditions were applied for all the leaves, the properties were different for CDs. The temperature in pyrolysis should neither be too high nor too low, as a lower temperature will result in incomplete carbonization while a high temperature will over oxidize the samples, ultimately resulting in deteriorated C-dots. Their surface structure will be destroyed at high temperature. The reaction time of C-dots’ synthesis has almost a similar effect as that of temperature, as higher reaction time will destroy the surface structure while lower temperature will lead to inappropriate morphology. The optical properties of CDs are temperature-dependent, i.e., the reaction time has a direct effect on the optical properties of CDs. Due to the non-availability of appropriate reaction temperature, the end product will end up with a useless one [137]. For the CDs obtained from different carbon sources and different methods, the influence of the pH value of the solution on their fluorescence emission intensities varies. The CDs have a wide range of pH workability. Some of the CDs work better at neutral pH while some work better at an acidic pH; some have optimal features at alkaline pH. Such types of CDs are suitable for pH sensor manufacturing, as they can withstand variations in pH. The CDs made from coconut husks have a wide range of functional groups on their surface which makes them suitable for making CDs for pH sensing. When there was an increase in pH from 4 to 12, there was protonation and deprotonation of carboxyl groups present. Consequently, there was a change in the electrostatic charging property and hence the fluorescence emission property of CDs decreased gradually.

There are several studies where heteroatom doping has been used in order to enhance the features of CDs. By introducing heteroatom in the framework of CDs, one can easily govern the electrical properties as well as chemical features associated with internal and surficial C-dots. C-dots are generally doped with N and S atoms, where the former is more electronegative than C, and the latter one regulates the energy state density of CDs. Due to S doping, there is an enhanced fluorescence emission intensity of C-dots [138].

The CDs are generally functionalized with various functional groups in order to enhance their fluorescence emission intensity, which as a result increases their applicability in the bioanalytical field. Due to surface functionalization, the surface defects will be decreased while exciton-hole recombination probability will be increased. Due to this surface passivation of CDs, there will be minimized or no agglomeration of CDs and enhanced fluorescence emission intensity [139].

## 5. Emerging Applications of Carbon Dots

**Figure 3:**
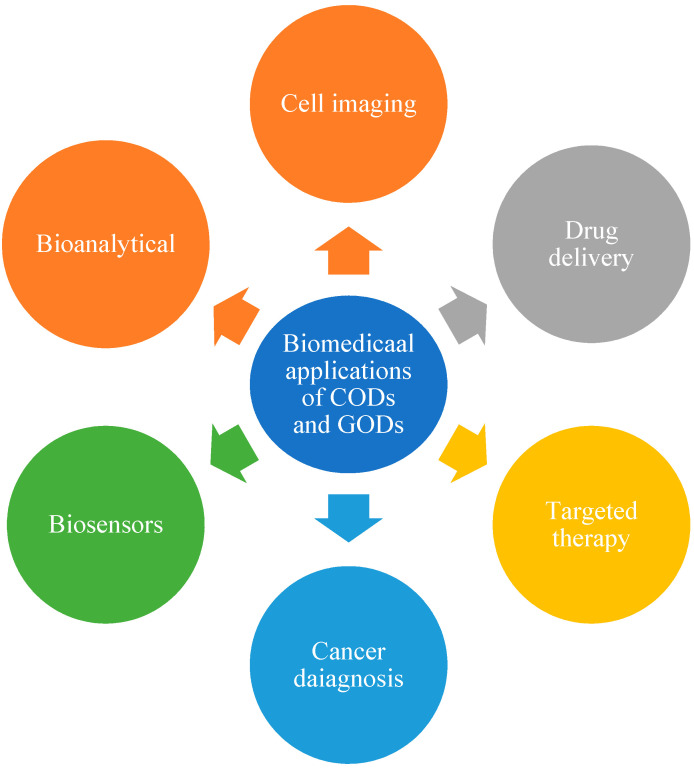
Biomedical applications of CODs and GODs.

**Figure 4:**
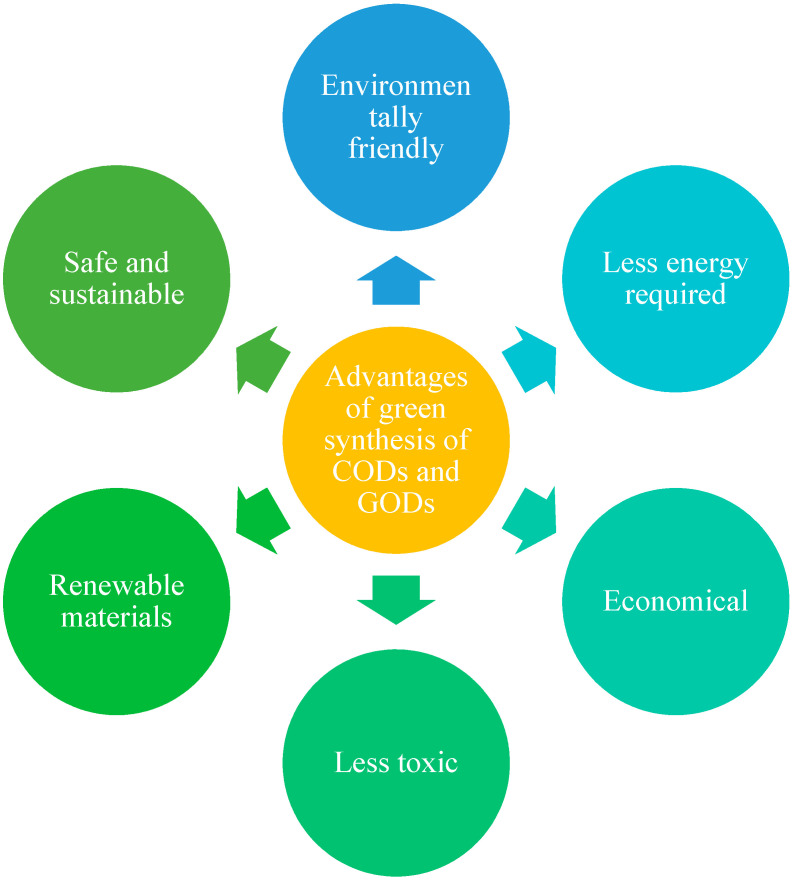
Advantages of green synthesis of CODs and GODs.

Although excellent properties such as low toxicity [140] and good biocompatibility make CQDs ideal materials for bioimaging, biosensors and drug delivery applications, CQDs can, however, also offer advantages in catalysis, sensors and optronics based on their excellent optical and electronic properties [123. The applications and advantages of CDs is given below in Figure 3 and Figure 4 respectively. 

### 5.1. Bioimaging

Bioimaging is a technique for imaging and direct observation of biological methods in real time which is often used to obtain information from the 3D morphology of the observed specimen from the outside, i.e., without physical interference [123]. Because of its fluorescence emissions and biocompatibility, CQDs are being used for bioimaging. In vivo images can be generated by injecting solvents carrying CQDs into a living organism for identification or treatment purposes. Organic-dye-conjugated CQDs are used as an efficient fluorescent H_2_S probe. H_2_S presence may change blue to the green emission of organic-dye-conjugated CQDs. Therefore, organic-dye-conjugated CQDs are able to reveal changes in the physiologically important rates of H_2_S using a fluorescence microscope. Some of the CDs have been used for the diagnosis of cancer and phototherapies. For instance, 200 µL of CQDs and wheat straw were given to the mice in the tail vein, and later on the fluorescence property was investigated. Zhang and Hong revealed the synthesis and mechanism of photoluminescence and their applications in our everyday life. Das et al. reported the use of carbon dots in the field of medicine and bioimaging [123]. Shabasy et al. also reported the detailed applications of C-dots in the biological field including bioimaging [140]. Several studies were carried out where CDs have been used for bioimaging; these are presented in Table 10.

### 5.2. Sensing

CQDs have also been used as biosensors for their versatility in alteration, high water solubility, nontoxicity, good photostability and excellent biocompatibility. Biosensors based on materials based on CQD and CQs may be used to track cellular copper, glucose, pH, H_2_O_2_ trace levels and nucleic acid visually. A useful application is lateral flow assays with nucleic acid. The detecting tags on the amplicons are identified by their associated antibodies and fluorescence patterns from the corresponding CQDs. The fluorescence of CQDs reacts effectively to pH, local polarity and the existence of metal ions in solution, which further extends their capacity for nanosensing applications, for example in pollutant analysis. Shabasy et al. and Das et al. reported the applications of carbon dots, especially quantum dots and graphene dots in the field of sensing. It has been used widely for the sensing of temperature, pH, light, phase, solvent, pressure and multisensitivity [123,140]. Several studies were carried out where CDs were used for sensing pH and organic and inorganic pollutants from water bodies; these are presented in Table 11.

### 5.3. Drug Delivery

CQDs’ nontoxicity and biocompatibility enable them to be used in wide-ranging applications in biomedicine, including drug carriers, fluorescent tracers and monitoring drug release. This is evidenced through the use of CQDs as photosensitizers in photodynamic therapy for cancer cell destruction. There are numerous examples in the literature where C-dots have been used for the delivery of numerous drugs in humans and animals. The major advantage of such C-dot-based drug delivery is that the carriers are nontoxic, PL and biocompatible in nature. Due to their small size and enhanced surface area, there is faster uptake of C-dots as a carrier by the cells. Consequently, there is a minimum adverse effect on the carrier molecule. It has been used successfully for the release of the doxorubicin drug. Doxorubicin conjugated with GODs exhibited a potent cytotoxic activity against U251 glioma cell GQDs through hydrogen bonds. C-dots have also been used for the delivery of SN38 by using PEGylated nanographene. By using graphene quantum dots, pancreatic cancer cells were investigated, along with biodegradable polyester vectors [140]. Several studies were carried out where CDs have been used for drug delivery; these are presented in Table 12.

### 5.4. Catalysis

Carbon dots (CDs) with sizes below 10 nm are found to be efficient catalysts or photocatalysts [154]. The versatility of surface modification towards different CQD groups enables them to absorb lights of various wavelengths, thus providing good prospects for photocatalytic applications. The P25 TiO_2_ composites modified by CQDs exhibited enhanced photocatalytic H_2_ evolution under UV-Vis irradiation. The CQDs act as a repository for electrons to increase separation efficiency of the P25 electron-hole pairs [156].

### 5.5. Optronics

CQDs have the ability to act as materials for dye-sensitized solar cells, organic solar cells, supercapacitors and tools for light pollution [157]. CQDs can be used as photosensitizers in dye-sensitized solar cells and the performance of photoelectric conversion is greatly enhanced. Hybrid silica-based sol embedded in CQD can be used as clear fluorescent paint [158]. The optical properties of the CQDs can be enhanced and improved by synthesizing multicolor-emitting CDs. It has been proven in the literature, theoretically, that the electronic features of carbon-based nanoparticles could be monitored by shape, by morphology, chemical composition and doping. In particular, CDs were considered as nanoparticles with sp^3^-hybridized amorphous carbon cores that contain partially sp^2^-hybridized carbon domains, and it was predicted that the emission of CDs can be red shifted by increase of hybridization factor of those domains within CDs [159]. Another theoretical investigation showed that covalent-bonded dimers of polyaromatic hydrocarbons at the CDs surface, in contrast to non-interacting monomers, resulted in a red shift and broadening of the PL band, with a decrease in its intensity [160]. CQDs are widely used in dye-sensitized solar cells, LEDS, photovoltaic cells, electroluminescent CD-based LEDs and CDs in Perovskite solar cells. Several studies were carried out where CDs were used for catalysis, photocatalysis optronics and in forensics; these are presented in Table 13.

### 5.6. Finger Print Recovery

The small size and reactive nature of CQDs make them highly suitable for visualization of latent fingerprints, due to which they can be easily bound with the ridge of fingerprints. One major advantage with the use of CQDs for latent fingerprinting is that there is no effect of aging of organic and inorganic content from the fingerprint samples. As with aging, the organic and inorganic content of fingerprints fades away and becomes difficult to detect for the investigators. Earlier, it was reported by Wang and his coworkers, that latent fingerprints can be collected using carbon dots made from pig intestines. They also found out that carbon dots imbedded in polyvinyl alcohol (PVA) due to long-term stability of films give a highly detailed image of the fingerprint. Recently, it has been seen that citric acid, which acts as a source of carbon with diethylenetriamine, gives good results. Carbon dots are mixed in liquid PVA and are cast on the fingerprints and the casted film is allowed to dry. After being dried, fingerprints can be visualized with UV flashlight within a frequency around 395–400 nm. G-CDs method, SiO_2_@ C-dot powder, N, S-SFCD method and carbon polymer dots (CPDs) starch powder were successfully used for the detection of latent fingerprints [165,166].

### 5.7. Antibacterial Activity of CDs

There are numerous examples from the literature where CDs have been used to control microbial activities. Among all the microorganisms, CDs have been widely used against viral diseases. Some of the investigators have revealed that CDs can interact with viruses and minimize the infection arising from them. Amino-acid- or boronic-acid-conjugated CDs have been used against the human herpes virus type-1 and showed that the entry of viruses was stopped [167]. In another study, phenylboronic-acid-conjugated CDs have shown potential against highly infectious virus diseases, such as coronavirus. Mechanistically, it may be due to the human coronavirus-229E entrance inhibition, caused by the interaction of the boronic acid functions of CQDs with the HCoV229E S protein through pseudo-lectin-based interactions [168]. Besides this, CDs have also been used for several bacterial pathogens, such as *Escherichia coli*, *Pseudomonas aeruginosa* and *Staphylococcus aureus,* which cause diarrhea, pus and burn infections and food poisoning and skin infections, respectively. The labelling of CDs helps in the fluorescence activity by which dead bacterial cells can be easily detected. These labelled CDs initially interact with the Gram-negative bacteria and then after adsorption on their surface exhibit fluorescence emission at higher intensity. The mode of action of such CDs is due to the imbalance in the surface charge and insertion of CDs into the surface by long alkyl chains which ultimately leads to the destruction of the bacterial cell wall and inhibition of bacteria. In one study, organosilane was loaded with quaternary ammonium compounds and exhibited antibacterial activity against Gram-positive bacteria. Here, glycerol was used as a source of carbon, due to its easy availability and economical nature [140,169].

Besides this, there are several studies that have been conducted by numerous scientists to expand and modulate the fluorescence properties of CDs, heteroatomic doping and surface functionalization, which were used to improve the physicochemical properties of CDs used in monitoring and antibacterial applications [170].

### 5.8. Applications of CDs in Chemical Warfare

Warfare agents (biological or chemical) have now become the first choice of every country against their enemies due to their greater impact and severity in comparison to physical means of mass destruction. *Bacillus anthracis (plague)*, *Francisella tularensis (tularemia), Yersinia pestis (plague)* and *Brucella abortus (brucellosis),* etc. have been used earlier as bioweapons either by armies or terrorists for the mass destruction of their opponents. Though government bodies have banned the use of such bioweapons, still some countries are using them secretly. Similarly, there are certain chemical compounds (phosgene, ricin, hydrogen cyanide, etc.) that are used in the place of bioweapons for the mass destruction of opponents’ countries or organizations. Most of these chemical warfare agents are colorless and could not be detected easily, so there is a need for highly sensitive sensors for the detection of such agents. By using such highly sensitive chemical sensors, one could prevent the mass destruction of an army or people at the earliest stage. There are several pieces of literature where CDs have been used as a warfare agent and also for the sensing of the harmful chemicals used during wars; for instance, Chang et al. used CD-chelated europium for the ratiometric fluorescent detection of biomarkers for biological warfare agents [25]. Kumar et al. developed a CNT-based gas sensing agent (NO_2_) for the study of simultaneous reversible and irreversible adsorption [27]. Tuccitto et al. synthesized a fluorescent nanosensor, based on carbon nanoparticles (CNPs), which was covalently functionalized with ethanolamine arms for interaction with nerve agents [171]. Again, Tuccitto et al. synthesized a fluorescent CDs nanochemosensor, for the selective detection of amino acids [26]. Tuccitto et al. also developed functionalized CDs for the supramolecular sensing of the chemical warfare agents’ simulants [28]. Similarly, Paul et al. used gold nanoparticles for the detection and degradation of chemical warfare stimulants [24]. Butera et al. also summarized the importance of CDs in supramolecular sensing of chemical warfare agents [23].

## 6. Surface Functionalization of CDs

Pathogenic bacteria monitoring using CDs can be categorized into two types, direct and indirect monitoring. Direct labeling and detection of pathogenic bacteria using CDs need different surface groups on the CDs or different surface charges, thus encouraging different bacteria to be labeled and detected [172]. Otis et al. presented CDs, used as precursors of citric acid and aminoguanidine. It may selectively mark strains of *Pseudomonas aeruginosa*. Codoped CDs (PNSCDs) with phosphorus, nitrogen and sulfur can selectively identify dead microbial cells but not live ones [173] [73]. Fluorescent CDs have been synthesized from bacteria and were used to mark dead microbial cells instead of live ones [174].

Indirect monitoring of pathogenic bacteria generally requires the application of CDs with a selective binding group to detect pathogenic bacteria, coupled with a labeling probe [175]. To couple the labeling probe, the surface group (e.g., amino group, carboxyl group or sulfhydryl group) of the CDs needs to be modified. Specific elements of identification, including antibodies, peptides and aptamers, stain and detect pathogenic bacteria. An inventive fluorescent aptasensor for the detection of *P. aeruginosa* was developed by graphene oxide quantum dots (GOQDs) [176]. Yang et al. prepared breakable organosilicon nano capsules (BONs) packaged with CDs and used the CDs-BONs to detect *S. aureus* in a fluorescent immunoassay.

Amides and amines of N-doped-CQD have played a significant role in improving antibacterial activity [177]. Electrostatic interactions between CQDs and bacterial surfaces were associated with their microbial killing mechanisms. A new approach for antimicrobial treatment is provided by amino terminal CDs (CDs-NH_2_) modified with ampicillin (AMP) [178]. The AMP-CDs and reactive oxygen species (ROS) produced under visible light irradiation are highly effective at preventing *E. coli’s* growth. Amine-functionalized lauryl-betaine-immobilized CDs provide antimicrobial properties, leading to the killing of Gram-positive (*S. aureus*).

## 7. Hetero Atomic Doping

The fluorescence features and the quantum yield (QY) of the CDs are vital for observing pathogenic bacteria. Heteroatomic doping is one of the most effective methods of fluorescence regulation and improvement of CD properties. This approach will alter their underlying electronic characteristics and give new active sites [14]. These were used in heteroatomic doping of CDs boron, magnesium, nitrogen, sulfur, phosphor, terbium Si, F, Se and metal ion (Mn^2+^, Fe^2+^, Co^2+^ and Ni^2+^). The synthesis of highly visible fluorescent phosphorus and nitrogen-codoped CDs (PNCDs) revealed that they could still act as an efficient and accurate fluorescent sample to identify the living state of microbial cells. PNSCDs can identify dead bacteria, selectively, though not live bacteria (Song et al. [179]). Carbon nitride doped with fluorine (F–C_3_N_4_) quantum dots were synthesized with such a high QY of 39.03 percent that was used, effectively, as a microbial imaging PL sensor [180]. Hojaghan et al. made extremely luminescent graphene doped with nitrogen quantum dots (N-GQDs) and used them to identify *S. aureus* and *E. coli* as a fluorescent probe [181].

N-doped-CD amides and amines played an immense role in enhancing their antibacterial activity. The ability of oxygen species to activate via the CD surface has a definite antibacterial effect [177]. CDs codoped with nitrogen and zinc (N, Zn-CDs) have demonstrated significant bacteriostatic activity against *E. coli*, which was 1 mg mL^−^^1^ with minimum inhibitory concentration (MIC). Jian et al. prepared spermidine-doped CDs with super cationic nitrogen that demonstrated effective antimicrobial activity against both sensitive multidrug-resistant bacteria [182]. Inspection of the proposed pathways showed that the super cationic N-CDs with highly positive charge result in severe breaking of the cell-membrane bacteria. Liu et al. used metronidazole as antibiotic to produce CQDs through a simple hydrothermal process. They produced a form of CQDs capable of detecting binding anaerobes through photoluminescence and this was successively effective against these bacteria [183]. Thakur et al. conjugated the antibiotic ciprofloxacin hydrochloride to the surface of CQDs made from gum Arabic in order to generate Cipro CQDs using a microwave-assisted synthesis process [184]. So, CQDs can also serve as carriers of drugs. Their study results revealed that Cipro CQDs have shown increased antimicrobial activity against the Gram-positive and Gram-negative bacteria tested [184]. Scott et al. used polyamines as the original organic raw material, and using simple methods of pyrolysis, super cationic carbon quantum dots (CQDs) could be rendered quickly. Since polyamines are natural products and the preparation method was green, these CQDs derived from polyamines show low toxicity and high biocompatibility; however, they possess high antibacterial activity. Shanka et al. synthesized CDs as a source of carbon from carbohydrate and cysteine (Cys) and o-phenylenediamine (OPD) as a source of nitrogen. CDs based on OPD have been more successful against *E. coli* with a rating of IC50 of ~200 μg/mL compared to Cys-based CDs while fluorescence emissions for later CDs were high. The hydrophilic CQDs were encapsulated in polyurethane polymer by Maria et al.; using simple swell-encapsulation-shrink Cyst/PU nanocomposite has also been revealed to be bactericidal towards *S. aureus* and *E. coli* after 1 h of BL irradiation at 470 nm. The use of biodegradable starch as precursor has been documented by Verma et al., using microwave-controlled synthesis of catalytic fluorescent carbon dots. The synthesized C-dots demonstrated catalytic activity in the Ag NPs’ photoreduction [185]. This treatment of Ag NPs and C-dots has been observed to be highly bactericidal, with substantially low silver concentration compared to Ag NPs. The composite was capable of penetrating the bacterial cell wall and resulted in higher ROS production levels. Hao et al. present a one-step electrochemical method of producing low-toxic and degradable carbon dots (CDs) using vitamin C. Such newly produced CDs possess high broad-spectrum antibacterial activity and antifungal activity even at low concentrations, although they break the bacterial walls during the diffuse entry, disrupt the secondary structures of bacteria and fungus DNA/RNAs and suppress essential gene expressions in order to ultimately kill the bacteria and fungus. Niu et al. have provided a one-step pyrolysis method to synthesize NCDs with excellent water solubility, good stability and 28% high quantum yield [186]. Due to the close interaction between the nitrogen-doped CDs and antibacterial drugs, the detection efficiency of NCDs for antibacterial drugs has been shown to effectively improve fluorescence [186]. Han et al. prepared cow-milk-derived carbon dots (CMCDs) which have been produced via hydrothermal cow milk treatment, and the as-prepared CMCDs have been removed by ethyl acetate to produce amphiphilic CMCDs (ACMCDs) [187]. A solvent casting method was used to create the novel ACMCD-Ag/polymethylmethacrylate nanocomposite antibacterial film. The nanocomposite antibacterial film is considered to be of great importance in applications because of its outstanding antibacterial, light-admitting and versatile properties [187]. Jin et al. demonstrated a one-step method to synthesize AgNPs. The surface characteristics of AgNPs might affect the stability of AgNPs characterized by various bactericidal effects. Such small AgNPs demonstrate stronger consistency in the culture medium which leads to excellent bactericidal activity which can fully inhibit *Escherichia coli* (*E. coli*) growth at a concentration of 150 μM of silver atoms [188].

The following are possible frameworks for AgNP antibacteria:

(i) AgNPs can be readily absorbed into *E. coli’s* surface and damage outer membrane permeability and fluidity [189].
(ii) Smaller-sized AgNPs (7.3 ± 1.0 and 6.1 ± 0.8 nm) that permeate the *E. coli* membrane to associate with the respiratory chain and DNA.
(iii) Ag+ release can trigger the death of *E. coli*.

## 8. Future Perspectives

Carbon dots are emerging nanomaterials in every field due to their unique and remarkable properties. Though they could be synthesized by various physical, chemical and biological methods, the emphasis has to be given to the biological route since it would be economical and eco-friendly. Besides this, the biologically synthesized carbon dots will be biocompatible, which will boost their application in the field of biomedicine, especially in drug delivery.

## 9. Conclusions

Carbon dots are a new type of carbon-based material that has exceptional physicochemical properties, due to which they are widely used in the fields of material science, environmental cleanup and medicine and have caught global attention in the last decade. The present review focuses on the recent advancement in the field of C-dots highlighting the various synthesis methodologies, the use of waste materials as carbon source and applications of C-dots in various fields. If the source material for carbon dots is waste carbon materials, then the carbon dots will be economical as well as eco-friendly. Out of various methods, the synthesis of carbon dots by the green route is a highly preferred approach as the green synthesis strategies will also minimize pollution in the form of solid waste from the environment. Several synthesis methods of C-dots are being reported till now but they are still simple and high yields remain a challenge to their large-scale preparations. In the future, C-dots will be potential candidates for targeted drug delivery applications as drug carriers because of their biocompatible nature and nontoxic effects and they will also potentially be used in the agricultural industries; they could be made more effective and specific by surface functionalization. To summarize the present review, C-dot development is still in the infancy stage and more research is needed in the field for real applications. 

## Figures and Tables

**Table 1:** Laser-ablation-based synthesis of carbon dots.

Source Material of Carbon	Size of C-Dots	Morphology	Applications	References
Graphite in polyethylenimine and ethylenediamine	A fairly spherical shape with diameters from 1 to 3 nm		Luminescent CD material for various applications	[42]
Graphite target irradiation	2–3 nm		Various potential associated applications	[43]
C-dot NPs functionalized with PEG200 and N-acetyl-l-cysteine	Thin (about 750 nm), homogenous and smooth (roughness of 2.7 ± 0.7 Å) film		Sensor for Hg (II) ions	[44]
Graphite powders	Ultrasmall size 1 nm		Sensing and catalytic applications	[45]
Graphite	Flowerlike morphology of the particles with a broad size distribution ranging from 200–500 nm.		Photoluminescence-ability-related applications	[46]
Mercapto-succinic acid				[47]
Carbon solid	Induced in acetone with laser pulses of 1064, 532 and 355 nm	Close-spherical amorphous CNDs with sizes between 5 and 20 nm	Light-emitting purposes	[48]

**Table 2:** Arc-discharge-based approaches for the synthesis of C-dots.

Scheme 52	Size/Shape of C-Dots	Applications	References
Carbon byproducts		Optoelectronic applications	[52]
TiO_2_ nanostructures coupled with carbon dots	Spherical shaped with an average size of 27 nm	Potential optical sensing applications	[53]
Boron-doped graphene quantum dots	Zero-dimensional graphene quantum dots (GQDs)	Potential luminescence and optical applications	[54]
CNTs and oxidized CNTs			[50]

**Table 3:** Electrochemical-based methods for the synthesis of C-dots.

Source Material of Carbon	Morphology of C-Dots	Applications	References
Sodium citrate and urea (by electrochemical carbonization)	Varies from 1.0 to 3.5 nm and with an average size of 2.4 nm (water soluble, fluorescent)	Sensing application of Hg^2+^ determination in real samples	[62]
Orange juice + in ethanol and after dichloromethane	Small-sized power preparation	Detection of neurotransmitters, using nontoxic reagents	[63]
MWCNTs in propylene carbonate	Green luminescent, (GQDs) with a uniform size of 3, 5 and 8.2 (±0.3) nm	Biomarkers, nanoelectronic devices and chemosensors	[64]
A carbon dots (CDs) and chitosan (CS) composite film modified by glassy carbon electrode	A fine power for electrode coating for composite formation	Biosensing of dopamine	[65]
HAuCl_4_ into carbon nanodots solution	Composite containing Au/carbon dot NC, graphene and a ferrocene derivative	Sensing and detection of ascorbic acid, dopamine, uric acid and acetaminophen	[66]
Fe_3_O_4_@MnO_2_ and N-doped carbon dots (NCDs).	Powdered fluorescence quencher and electrochemical enhancer material	Determination of hydrogen peroxide	[67]

**Table 4:** Microwave-assisted synthesis of C-dots.

Source Material of Carbon	Size/Shape of C-Dots	Applications	References
Sucrose as C source, and diethylene glycol (DEG) as the reaction medium	Water dispersive and transparent with average diameter about 5 nm	Optical nanoprobing	[76]
Chitosan (1%) solution + acetic acid (1%) solution, addition of CDs to obtain carbon dots-chitosan (CD-CH) composite.	A thin film of CD-CH composite was prepared on ITO glass substrate	Sensing of vitamin D_2_	[77]
N-phosphonomethyl aminodiacetic acid and ethylenediamine (thermolysis + microwave)	Uniform dispersion with the average size of 3.3 nm	Cellular imaging	[78]
Citric acid, urea and thiourea	~10 nm	Detection of Hg^2+^ and I^-^ in tap, river and mineral waters and fish samples	[79]
Cystine as source for C, N and S and glycerol as the reaction solvent	Fine-sized powder	Detecting Hg(II) in spiked tap and lake waters	[80]
From lactose by microwave-assisted hydrochloric acid	Average size 10 nm (fluorescent water-soluble carbon dots (CDs))	Analysis of various heterocyclic aromatic amines	[81]
Citric acid was used and glutathione or thiourea as precursors of doping elements	Simple fine powder	Mercury in river water and wastewater	[82]
Kelp (algae) as main carbon source and ethylenediamine as nitrogen dopant	Below 10 nm	Detection of Co^2+^ based on fluorescence quenching	[83]

**Table 5:** Thermal-decomposition-based synthesis of C-dots.

Source Material of Carbon	Synthesis Conditions	Size/Shape of C-Dots	Applications	References
Ascorbic acid (AA) and uric acid (UA)	Electrochemical method	Fine carbon fiber electrode	Highly sensitive and selective dopamine (DA) detection	[86]
Carbon dots (CDs)/g-C3N4/ZnO nanocomposite	Facile impregnation-thermal method	Composite by using impregnation-thermal method	Tetracycline photocatalysis in the water environment	[87]
Citric acid monohydrate, as carbon precursor solid-phase composite of CDs deposited on graphene	Carbonization+ microwave	CD–graphene-interaction-based nanomaterial 1–10 nm	Light conversation applications	[88]
Gadolinium-doped carbon dots (Gd-doped CDs)		~18 nm with dispersibility in water	MRI-guided radiotherapy of tumors	[89]
Citric acid	240 °C for only 1 min	~0.9 nm		[90]
Citric acid	200 °C for 30 min	0.7–1 nm		[91]
1-butyl 3-methyl imidazolium bromide and L -cysteine	240 °C	1.0–3.5 nm		[92]
Citric acid and dicyandiamide		Spherically shaped		[93]

**Table 6:** Carbonization-based methods for synthesis of C-dots.

Source Material of Carbon	Size of C-Dots	Applications	References
L-cysteine+ citric acid	Near-spherical particles with diameters in the range of 2–4 nm, crystalline	Fluorescent agent for cell imaging	[96]
Citric acid	4.8–9 nm		[97]
6-*O*-(*O*-*O*-dilauroyl-tartaryl)- D -glucose	~ 2.4 ± 0.5 nm		[95]

**Table 7:** Pyrolysis-based methods for synthesis of C-dots.

Source Material of Carbon	Size of C-Dots	References
L-aspartic acid and D-glucose	Diameter of CDs was 2.28 ± 0.42 nm	[102]
Citric acid via thermal pyrolysis method, capping agent: diethylenetriamine	CDs ranged from 5 to 8 nm	[101]
Tris base, gadopentetic acid and betaine hydrochloride	~3.2 nm	[99]

**Table 8:** Synthesis of C-dots by the solvothermal method.

Source Material of Carbon	Hydrothermal Conditions	Size of C-Dots	Applications	References
Wheat bran	180 °C, 3 h		Drug delivery	[105]
Tartaric acid and bran	Autoclave at 150 °C for 8 h in the oven	~4.85 nm	G-CQDs were used as a fluorescent probe for detection of Cu^2+^ ions	[106]
Orange peels	180 °C, 12 h		LEDs, photocatalysis	[107]
Cereals and grains waste	200 °C, 12 h		Imaging, sensing, labeling	
Bamboo waste	200 °C, 6 h		Bioimaging (in vivo)	[108]
Lemon peels	200 °C, 12 h		Sensing and photocatalytic	[109]
Coconut husks	200 °C, 3 h		pH sensing	[110]
Sugarcane bagasse	190 °C, 24 h		Drug delivery	[14]
Prawn shells	180 °C, 12 h		Nitrite detection	[14,111]
Wheat straw	250 °C, 10 h		-	[112]
Carbon tetra chloride		~3.3 nm	-	[103]
SiCl_4_ and hydroquinone	SS autoclave at 200 °C for 2 h	7 ± 2 nm	-	[104]
Hydroquinone and boron source BBr_3_		~16 nm	-	[113]
Low-cost wastes of willow bark	Hydrothermal + carbonization		Glucose detection	[114]

**Table 9:** Synthesis of C-dots by the ultrasonic method.

Source Material of Carbon	Size of C-Dots	Applications	References
Activated carbon	5–10 nm	-	[117]
Activated carbon	5–10 nm	-	[121]

**Table 10:** Applications of C-dots in bioimaging.

Source Material of Carbon	Methods of Synthesis	Applications	References
Onion waste	Hydrothermal	Multicolor imaging and Fe3+ detection	[141]
Lychee waste	Solvohydrothermal	Multicolor cell imaging and Fe3+ detection	[141]
Cow dung waste	Chemical oxidation	Live-cell imaging with subcellular selectivity	[142]
Banana peel waste	Hydrothermal	In vivo bioimaging	[143]
Walnut shells	Carbonization	Intracellular bioimaging	[144]
Wheat straw and cereals	Hydrothermal	Cell imaging and in vivo bioimaging	[112]

**Table 11:** Applications of C-dots in the sensing field.

Source Material of Carbon	Morphology of C-Dots	Applications	References
Bagasse waste	Hydrothermal (HT)	Hg^2+^ detection	[145]
Lignocellulose waste		Copper ion	[146]
Crown daisy leaf waste		Copper ion	[147]
*Sargassum fluitans*		DNA detection	[148]
Mango peels waste		Mesotrione detection	[124]
Glucose source		Sensing	[123,140]
Palm shell waste	Ultrasonic	Nitrophenol detection	[149]
Waste tea residue	Chemical oxidation	Tetracycline detection	[14]
Waste candle soot		Hg^2+^ and Fe^3+^ detection	[150]
Kerosene fuel soot		Picric acid, Fe^3+^ and Cu^2+^ detection	[151]

**Table 12:** Applications of C-dots in drug delivery.

Source Material of Carbon	Methods	Applications	References
Wheat bran	HT	DD	[105]
Sugarcane bagasse	Burning and HT	DD	[152]
Waste sago bark	Catalyst-free pyrolysis	Anticancer drug delivery	[153]
Bamboo leaves	Reflux	DD, tumor imaging	[154]
Crab shells	Microwave	DD	[155]

**Table 13:** Applications of C-dots in catalysis, optronics and forensics.

Source Material of Carbon	Methods	Applications	References
Waste frying oil	HT	Photocatalyst	[14]
Citrus fruits peel waste (lemon + orange)	HT	Photocatalyst and sensing	[161]
Lignocellulose waste	Pyrolysis	Photocatalyst attached to pollutant utilization	[162]
Bitter apple waste	Pyrolysis	Photocatalyst	[163]
Waste food	HT	Light-emitting diodes	[164]

## Data Availability

Not applicable.

## Abbreviations

2-AP	2-aminopyridine
AA	Ascorbic acid
AcD	Arc discharge
AEAPMS	N-(β-aminoethyl)-γ-aminopropyl methyl dimethoxysilane
AFM	Atomic force microscopy
Ag	Silver element
AgNP	Silver nanoparticles
AMP	Ampicillin
Au	Gold
BBr_3_	Boron tribromide
Ca	Calcium
Ca	Citric acid monohydrate
Cd	Cadmium
CD or C-dots	Carbon dots
CD-CH	Carbon dots-chitosan
Cm	Centimeters
CMCDs	Cow-milk-derived carbon dots
CNPs	Carbon nanoparticles
CNTs	Carbon nanotubes
CQD	Crystalline carbon core
Cu	Copper
Cys	Cysteine
DD	Drug discovery
DEG	Diethylene glycol
DMF	Dimethylformamide
DNA	Deoxyribonucleic acid
ECE	Electrochemical exfoliation
FCD	Fluorescent carbon dots
Fe_3_O_4_	Iron oxide
FG	Functional groups
HAuCl_4_	Hydrogen tetrachloroaurate(III) hydrate
Hg	Mercury
HPPT	4-hydroxy-1H-pyrrolo[3,4-c]pyridine-1,3,6(2H,5H)-trione
HT	Hydrothermal
IPCA	Imidazo[1,2-a]pyridine-7-carboxylic acid
LA	Laser ablation
LED	Light-emitting diode
MIC	Minimum inhibitory concentration
MnO_2_	Manganese dioxide
MWCNTs	Multi-walled carbon nanotubes
NaNH_2_	Sodium amide
NaOH	Sodium hydroxide
Ni	Nickel
Nm	Nanometers
NPs	Nanoparticles
NR-CDs	Nitrogen-rich blue, fluorescent carbon dots
OPD	O-phenylenediamine
PAHs	Polycyclic aromatic hydrocarbons
PD	Polymer dots
PEG	Polyethylene glycol
pH	Potential of hydrogen
PL	Photoluminescence
PNSCDs	*Pseudomonas aeruginosa*
PPEIE	Poly (propionyl ethylene-imine-ethyleneimine)
PPEI-EI	Poly (propionylethyleneimine-co-ethyleneimine
Pt	Platinum
PVA	Polyvinyl alcohol
QCE	Quantum confinement effect
QD	Quantum dots
QY	Quantum yield
RB	Rose Bengal (RB)
ROS	Reactive oxygen species
Rpm	Rotations per minute
SC	Sodium citrate
Se	Selenium
Si	Silicon
SiCl_4_	Silicon tetrachloride
SWCNTs	Single-wall carbon nanotubes
Tarp	Tryptophan
TEM	Transmission electron microscopy
TETA	Triethylenetetramine
TiO_2_	Titanium dioxide
TTDDA	4, 7, 10-trioxa-1,13-tridecanediamine
UA	Uric acid
UV-Vis	Ultraviolet visible light
XRD	X-ray diffraction
Zn	Zinc
